# Autonomous IoT Monitoring Matching Spectral Artificial Light Manipulation for Horticulture

**DOI:** 10.3390/s22114046

**Published:** 2022-05-26

**Authors:** Irene Cappelli, Ada Fort, Alessandro Pozzebon, Marco Tani, Nicola Trivellin, Valerio Vignoli, Mara Bruzzi

**Affiliations:** 1 Department of Information Engineering and Mathematics, University of Siena, 53100 Siena, Italy; ada@diism.unisi.it (A.F.); tani@diism.unisi.it (M.T.); vignoli@diism.unisi.it (V.V.); 2 Department of Information Engineering, University of Padova, 35131 Padova, Italy; alessandro.pozzebon@unipd.it; 3 Department of Industrial Engineering, University of Padova, 35131 Padova, Italy; nicola.trivellin@unipd.it; 4 Department of Physics and Astronomy, University of Florence, 50019 Florence, Italy; mara.bruzzi@unifi.it

**Keywords:** photovoltaic, solar cells, energy harvesting, LoRaWAN, IoT, horticulture, renewable energy, environmental safeguard

## Abstract

This paper aims at demonstrating the energy self-sufficiency of a LoRaWAN-based sensor node for monitoring environmental parameters exploiting energy harvesting directly coming from the artificial light used in indoor horticulture. A portable polycrystalline silicon module is used to charge a Li-Po battery, employed as the power reserve of a wireless sensor node able to accurately monitor, with a 1-h period, both the physical quantities most relevant for the application, i.e., humidity, temperature and pressure, and the chemical quantities, i.e., O_2_ and CO_2_ concentrations. To this aim, the node also hosts a power-hungry NDIR sensor. Two programmable light sources were used to emulate the actual lighting conditions of greenhouses, and to prove the effectiveness of the designed autonomous system: a LED-based custom designed solar simulator and a commercial LED light especially thought for plant cultivation purposes in greenhouses. Different lighting conditions used in indoor horticulture to enhance different plant growth phases, obtained as combinations of blue, red, far-red and white spectra, were tested by field tests of the sensor node. The energy self-sufficiency of the system was demonstrated by monitoring the charging/discharging trend of the Li-Po battery. Best results are obtained when white artificial light is mixed with the far-red component, closest to the polycrystalline silicon spectral response peak.

## 1. Introduction

The number of wireless sensing systems and smart devices employed either in the internet of things (IoT) or in the wireless sensor network (WSN) frameworks is rapidly expanding. The need to have power autonomous devices or at most to improve their energy efficiency has become urgent, also in sight of expanding their average lifetime. In this context, light energy harvesting can represent a valid alternative to the usage of disposable batteries and to the exploitation of the power grid, which is not always an available or affordable solution. To this aim, several studies have been conducted for the design of photovoltaic (PV) modules suitable either for diffuse or direct sunlight and for artificial light sources [1,2,3]. Most of the indoor deployments exploit white lights but in certain applications different spectral compositions can be required. This is the case of indoor horticulture, which exploits ad hoc light treatments to improve plant growth, boosting the crop yield and promoting the development of specific morphological characteristics of plants. Moreover, light quality management concerns also the investigation of the effects of the different spectral components on the plant growth, allowing us to tailor the light source on the specific application. This also leads to water and energy saving and to the reduction in the use of chemical growth regulators [4]. The more suitable artificial light sources are Light Emitting Diodes (LEDs), which are very versatile since they feature several possible spectral distributions in the visible range, depending on their color temperature (CCT) and color rendering index (CRI).

Nowadays, most fruit and vegetable production is carried out in greenhouses, as they allow for controlled temperatures and humidity, functional to boost plant development and fruit production. In this context, the advent of IoT architectures and distributed monitoring systems represents a viable solution to integrate traditional greenhouses with sensors, actuators and wireless connectivity, with the aim of maintaining stable environmental conditions, by collecting sensors parameters and making them available for control tasks. The set-up of distributed sensor networks in the horticulture domain is further favored by the possibility of deploying energy self-sufficient sensor nodes, provisioning energy from the artificial light sources. The good compromise between data transmission coverage, cost effectiveness and low power requirement makes the IoT infrastructures dominant in several scenarios, from environmental monitoring [5] to the industrial field [6]. In all these contexts, data transmission is a crucial task and should be carefully evaluated. Local area technologies are widely adopted nowadays: while WiFi is not the best option due to its relatively high power consumption, Bluetooth or ZigBee may be ideal solutions. However, these technologies have maximum transmission ranges in the order of some hundreds of meters: these features are not suitable for monitoring structures intended for large greenhouse installations where the constructions can be hundreds of meters long and account for several greenhouses. Mesh networking may be implemented; however, such a layout requires continuously listening router nodes, critical again for power consumption. Conversely, the large-scale distributed monitoring task can be achieved with the so-called low power wide area network (LPWAN) transmission technologies. Among them, the long range (LoRa) one, implementing the associated LoRa wide area network (LoRaWAN) protocol, has the primacy thanks to the wide transmission range, the low power consumption and the satisfactory decoding capacity even in presence of critical noise and attenuation in the radio channel. Several contributions can be found in literature about the setting of data acquisition systems exploiting LoRaWAN transmission technology [7,8], including the horticultural field [9]. However, although this technology is designed for low power applications, battery replacement remains one of the primary bottlenecks in the sensor network deployment, especially in the case of a huge number of nodes located in remote sites. For this reason, great attention has been paid to the identification of energy harvesting solutions in the LPWAN domain capable of ensuring the permanent energy self-sufficiency of the nodes [10].

In general, environmental safeguard issues must include as a priority an efficient energy management. In this respect, a PV energy harvesting system directly using the artificial lighting may be an ideal solution. Nonetheless, spectral response of PV modules is optimized for Sun spectral distribution and Sun intensity [11]. However, indoor photovoltaic modules must work with artificial light spectra and intensities very different from those of Sun. Indeed, the ability of PV modules to efficiently work indoor to get energy-autonomous wireless sensing systems is nowadays still a vastly unexplored issue. In this respect, greenhouses represent quite a stimulating scenario, due to the extremely versatile spectral modulations adopted and their need to monitor a multiplicity of environmental parameters in conditions where grid connections may be difficult to be pervasively settled.

This paper focuses on the development and testing of a LoRaWAN-based sensor node equipped with a recharging system based on a polycrystalline silicon PV module and aimed at monitoring temperature, relative humidity (RH), pressure and CO_2_ and O_2_ concentrations. This novel sensor node has been tested under six lighting treatments commonly employed in the indoor horticulture field and containing spectral components in the blue, red, far-red and white 5700 K ranges.

The investigation was conducted by characterizing the module and testing the node under two programmable LED lights, one of which is a commercial lamp commonly used in greenhouses.

The rest of the paper is organized as follows. In Section 2 and Section 3, respectively, the state of the art about artificial lighting in horticulture and greenhouse monitoring is presented. In Section 4, we illustrate the materials and methods used, while Section 5 describes the node architecture. In Section 6, the experimental results of the PV module characterization and of the tests performed on the node are reported, also discussing the outcome of the tests. The conclusions close the article.

## 2. Artificial Lighting in Horticulture

The usage of artificial lighting in horticulture, both as exclusive light source and as a supplement to sunlight, is nowadays a trend topic since there is an increasing interest towards the thorough study of lighting protocols aimed at increasing the crop yield, speeding up the plant growth and performing an efficient use of water and nutrients. The plants’ photosynthetic reaction is developed by radiation in the photosynthetically active radiation (PAR) region, which includes the wavelengths in the [400 nm, 700 nm] spectral range [12]. However, radiation is not well absorbed by chlorophyll in the overall PAR region. Indeed, the relative quantum photosynthetic efficiency curve presents a drop of efficiency between 400 nm and 600 nm [13], corresponding to the green spectral contributions which are less capable to effectively produce photosynthetic reactions in plants. Most of the morphogenesis reactions are triggered by radiations in the blue, in the red or in the far-red spectral regions, in correspondence with the light absorption peaks, whilst green and yellow components have a minor effect on plant photomorphogenesis and a different participation on photosynthesis. Light is fundamental in the development and growth of plants not only because it provides energy for photosynthesis, but also because it influences the structural characteristics and the physiological responses of plants [14,15,16,17,18]. The type of used light source influences the development of the plant in different and not easily predictable ways since each spectral component acts differently on stem elongation, foliar expansion, roots development, nutrients concentration, etc. Moreover, not only are these interactions complex in themselves, but they may also vary depending on the plant species studied.

The red light ([625 nm, 720 nm] range) is fundamental for plant growth as its presence is sufficient to favor the normal photosynthesis even in the absence of the other spectral components [13]. The far-red light ([720 nm, 800 nm] range) as a sole light source is unable to establish photosynthesis in the plant as it is outside the wavelength range defining the PAR. However, in conjunction with other spectral components such as the red, blue and white ones, it favors the development of some plant’s characteristics. In particular, different red:far-red ratios (R:FR) influence the growth and the functionality of the plants in various manners. For example, low R:FR accelerates stem elongation, foliar expansion and flowering to the detriment of branching, which appears dampened [4]. Additionally, the blue light ([445 nm, 485 nm] range), used as a supplement to other spectral components, has positive effects on plant growth and nutrient concentration.

The remarkable technological developments of LEDs in recent years and also their availability in narrow spectral solutions have favored the primacy of this technology in the horticulture sector over other light sources, such as fluorescent lamps. Compared to traditional lamps, they have greater energy efficiency, longer lifetime (~50,000 h), low heat generation and important advantages from the point of view of the user and environmental safety, since they do not contain hazardous materials such as mercury, present in fluorescent lamps. Moreover, the small LED size and the low radiant heat allow the realization of versatile lighting solutions, such as inter-lighting arrangements where the light source is placed within the plant canopy in order to improve the light distribution for those crops characterized by very long stems along which the fruits grow. Furthermore, offering the possibility to choose only one specific wavelength, LEDs guarantee to not waste energy for spectral components less significant for the photomorphogenesis and the photosynthesis of the plant.

Commercial LEDs for indoor horticultural applications are specifically designed to feature Spectral Power Distribution (SPD) in the PAR range and the power they deliver is generally measured in terms of Photosynthetic Photon Flux Density (PPFD) [µmol/m^2^ s] [13]. These light sources are usually composed of LEDs arranged in array or matrix structures depending on the geometry and the dimensions of the area to be illuminated. Depending on the required spectrum, as long as it belongs to the PAR, the LEDs employed can have either the same or different peak emission wavelengths. These lamps are often provided with an ad hoc software, specifically thought to allow the modification of the intensity and the composition of the light spectrum, offering the user the possibility to implement several lighting programs with distinct photoperiods.

However, light source composition, illumination cycles and total amount of PPFDs delivered to the crop are factors strongly dependent on the cultivated plant species. Furthermore, in general, different lighting treatments are found to better stimulate certain plant characteristics while simultaneously worsening others. Therefore, there is no general lighting program valid for each type of cultivation, but it must be tailored to the specific application. In any case, the typical light intensity is in the range from 100 µmol/m^2^ s to 300 µmol/m^2^ s, whilst typical photoperiods are 12 hd^−1^, 16 hd^−1^ or 20 hd^−1^. Concerning the light spectrum composition, blue, red and far-red components are often supplemented with a white light in order to enhance the plant growth [16] or combined together in different ratios [14,19,20,21,22].

In [20,21], the authors investigate the effect of different red and blue spectral fractions and different PPFDs on indoor cultivation of lettuce and basil, in order to find the lighting treatment giving the best compromise between crop yield, nutrient content and light use efficiency. The scientific contributions in [14,16] present results obtained by adding the far-red radiation to the lighting treatments, both mixed with red and blue components [14], or as a supplement to white light [16]. In both cases, the papers demonstrate that the lighting protocols containing the far-red radiation promote the plant growth, determining higher yield in comparison with the other illumination programs.

## 3. Greenhouse Monitoring

The use of intelligent systems in the food production chain is nowadays seen as crucial to increase production levels, improve the quality of the products and reduce the environmental impact of the production cycles. While large efforts have been devoted to the definition of real-time monitoring and automation systems for outdoor crops [23], several contributions have also focused on the definition of technological infrastructures for greenhouses [24], where the application requirements are different in terms of scale and complexity, but still pose significant challenges, for example in terms of energy self-sufficiency of the nodes.

Since the emergence of wireless sensor network (WSN) technologies, distributed data collection infrastructures in charge of remotely transmitting different sets of environmental parameters in greenhouses have been extensively proposed. A comprehensive review, focusing on possible parameters to be monitored as well as on the different data transmission technologies to be employed in agriculture, is presented in [25]. While this paper deals in general with applications in agriculture, together with horticulture and aquaculture, greenhouses are also taken into account. Another detailed review of the state of the art, focusing on greenhouses in particular, is presented in [26]. In this work, the most significant parameters to be monitored in a greenhouse are listed (i.e., soil moisture and temperature, air humidity, air quality index, temperature, light intensity and CO_2_) as well as the main transmission technologies, discussing for each one the advantages and drawbacks. Moreover, a set of possible objectives of the monitoring infrastructures are analyzed.

While most of the contributions available in the literature propose systems that implement subsets of the features described in [26], a wide range of different architectures have been implemented. A first simple contribution is presented in [27], where a remote sensor node was designed to transmit soil moisture by means of 2.4 GHz IEEE 802.15.4 MICAz (Crossbow, San Jose, CA, USA) radio modules, while a more complex IEEE 802.15.4 WSN, implementing 6LowPAN protocol, is presented in [28]. In this work, several parameters are collected and transmitted, including relative humidity, temperature, luminosity and CO_2_ concentration. While no detail is provided concerning the power source of the sensor node in the first contribution, in [28], 1.5 V AA batteries are used to power the node.

A wide range of architectures based on the use of IEEE 802.15.4 2.4 GHz radio modules, in some cases exploiting ZigBee protocol, have been subsequently presented in the literature [29,30,31]. While using the same data transmission technology, all these solutions differ for what concerns the parameters to be monitored, the overall nodes architecture or the data collection and storage system. Laura et al. [29] propose the integration of a wide range of sensors, suggesting the monitoring of the leaves of the plants. Ting at al. [30] focus on the exploitation of the collected data for the implementation of neural networks to predict photosynthetic rate while Ferentinos et al. [31] show how data collected by means of WSNs can be used to estimate the spatial distribution of the environmental conditions inside a greenhouse, with the aim of identifying possible critical conditions for plants.

With the explosion of IoT technologies, new solutions for greenhouse monitoring have emerged, exploiting more efficient data transmission techniques, both in terms of energy consumption and of network coverage, as well as more complex data management architectures. Exploited data transmission technologies include UHF RFID [32], Bluetooth Low Energy [33] as well as LoRa [34], while some contributions propose the adoption of cloud services such as Thingspeak for data management [9,35]. Despite the great interest in setting up autonomous IoT infrastructures, little interest has been put on the problem of power management which may be in some cases an actual bottleneck. Indeed, the deployment of a large number of nodes may require either the setting up of a wired connection to the power grid or the use of a large number of batteries that may require periodic replacement. For this reason, the identification of a solution for the autonomous operation of the nodes is relevant for the actual implementation of deploy-and-forget systems. However, despite this importance, no contribution proposing the adoption of energy harvesting techniques in this context has been found in the literature. Thus, to our knowledge, the present study is the first work analyzing in detail the usage of a harvesting technology, i.e., polycrystalline silicon photovoltaic cells exploiting the artificial light, for the powering of indoor sensor nodes in greenhouses.

## 4. Materials and Methods

In this Section, the instruments and the procedures used to perform the PV module characterization and the node tests are presented.

### 4.1. Light Sources

The polysilicon photovoltaic module is characterized under two programmable light sources.

The first light source is a LED-based custom designed solar simulator with tailored spectral emission to match the requirement of IEC 60904-9. The emitters are high power LEDs which are subdivided in 6 spectral peaks: 460 nm, 530 nm, 630 nm, 780 nm, 850 nm and 940 nm. The lamp is composed of a matrix of 9 × 9 cells, each cell is constituted of a 3 × 2 LED structure (one LED for each wavelength) for a total of 486 uniformly distributed LEDs into a 300 mm × 300 mm light emitting surface. The 486 LEDs are subdivided in 3 concentric zones: central zone, middle zone and perimeter zone, each zone is controlled independently to ensure the irradiance uniformity on the target surface. The electrical configuration is then composed of a total of 18 LED channels. To ensure the temporal stability, each LED is driven by an independent constant current driver, the reference current is set by a DAC (Digital to Analog Converter) with a maximum value of 1 A; a microcontroller unit (MCU) then sets the DAC and communicates with a LabVIEW 20.0.1 software to control and monitor the 18 channels. The light source is able to reach an irradiance in excess of 1000 W/m^2^ s with a class A spectral match (according to IEC 60904-9).

The second light source is the FL384 by Flytech (Alpago, Belluno, Italy), a commercial solution specifically designed for the deployment of plant growth systems in indoor environments and in greenhouses. It is a flat panel with maximum absorbed power equals to 90 W, composed of 10 × 5 cells arranged in a matrix form, each cell has 4 LEDs for a total of 200 LEDs (50 for each channel) occupying a 22.3 cm × 50.0 cm active area. The 4 LED channels are peaked in different wavelengths ranges: [445 nm, 455 nm] (blue, B), [655 nm, 670 nm] (red, R), [720 nm, 750 nm] (far-red, FR) and white 3000 K (W) and each channel can be dimmed via software on a daily schedule. The dimming is performed by modifying the duty cycle of the signal driving the LEDs (250 Hz) and this requires filtering the cell output signal in order to stabilize it.

### 4.2. PV Module Characterization

A Keithley (Solon, OH, USA) 2401 source-electrometer is used to measure the current–voltage (I-V) and power–voltage (P-V) characteristics of the solar cells embedded in the node. A Pt100 temperature sensor is added to the automatized measurement chain, driven by PC through National Instruments and MATLAB 2021 Toolkits.

A Kipp & Zonen (Delft, The Netherlands) CMP3 pyranometer is used to measure the power density in the [300 nm, 2800 nm] spectral range impinging on the PV module (nominal sensitivity 15.66 µV/(W/m^2^ s)). It features a 64-junction thermopile detector and a 4 mm glass dome.

The spectral irradiance emitted from the light source is measured by means of a cosine corrected spectrometer, Ocean Insight (Orlando, FL, USA) USB4000 UV-VIS, equipped with a CC3 UV-S cosine corrector, by Ocean Insight too.

### 4.3. Node Tests

During the field tests, the charge status of the rechargeable Li-Po battery is monitored through the acquisition of its voltage level V_Li–Po_, continuously monitored with sampling period of 1 s by using an Agilent (Santa Clara, CA, USA) 34410A multimeter (6 ½ digit resolution) controlled via LabVIEW 20.0.1.

### 4.4. Light Treatments

The preliminary lighting test conditions are selected to emulate real situations in which a white light source is integrated with B, R or FR components, generally boosting the plant growth [16]. In particular, 3 test conditions are obtained by adding to the white light (5700 K) the same amount of blue, red and far-red PPFD and considering a 20 hd^−1^ photoperiod. These three lighting protocols are obtained as follows, giving a total amount of PPFD equal to 155 µmol/m^2^ s:

- WB: 117 µmol/m^2^ s of white 5700 K and 38 µmol/m^2^ s of blue (corresponding, respectively, to 75% and 25% relative intensities);
- WR: 117 µmol/m^2^ s of white 5700 K and 38 µmol/m^2^ s of red (corresponding, respectively, to 75% and 25% relative intensities);
- WFR: 117 µmol/m^2^ s of white 5700 K and 38 µmol/m^2^ s of far-red (corresponding, respectively, to 75% and 25% relative intensities).

Afterwards, three additional test conditions are used, in which no white light is employed and the light spectra are composed only by the combination of blue, red and far-red components, as proposed in [14,21]. In particular, the lamp is programmed with three different lighting treatments with a 20 hd^−1^ photoperiod and always supplying a total PPFD of 155 µmol/m^2^ s:

- BRFR: 17 µmol/m^2^ s of blue, 112 µmol/m^2^ s of red, 16 µmol/m^2^ s of far-red (corresponding, respectively, to 11%, 79% and 10% relative intensities);
- BR1: 25 µmol/m^2^ s of blue, 130 µmol/m^2^ s of red (corresponding, respectively, to 16% and 84% relative intensities);
- BR2: 38 µmol/m^2^ s of blue, 117 µmol/m^2^ s of red (corresponding, respectively, to 25% and 75% relative intensities).

**Figure 1:**
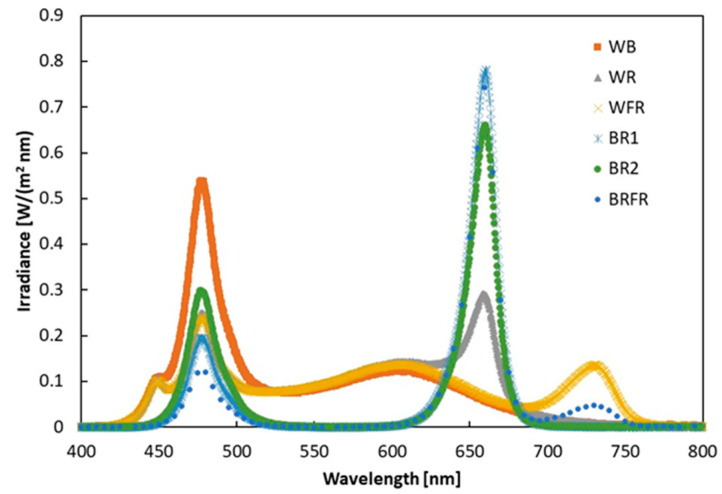
Spectral distributions measured by the spectrometer in case of the six studied spectral compositions; the integral of each spectrum corresponds to the intensity measured by the pyranometer.

The tested spectral distributions for each lighting condition are shown in Figure 1. The spectra have been measured with the spectrometer placed at the same location used for the PV module. The integral of each spectrum corresponds to the intensity measured by the pyranometer.

## 5. Node Architecture

**Figure 2:**
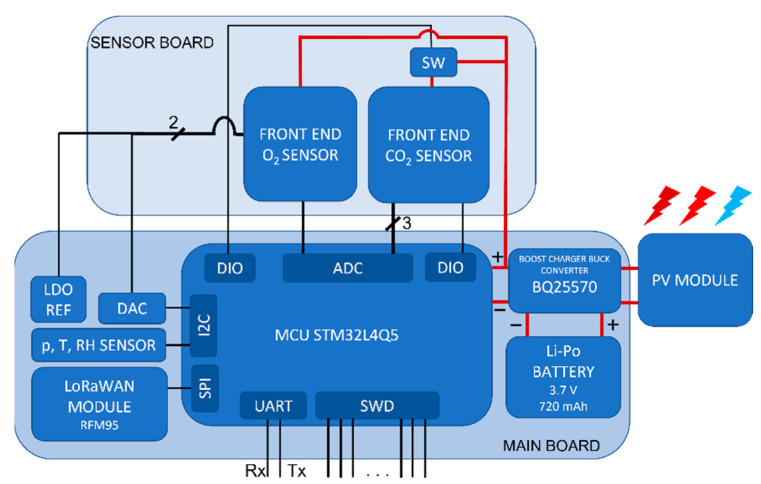
Architecture of the node: the main board, the sensor board and the PV module.

The node proposed in this work, whose architecture is shown in Figure 2, is designed to measure with a satisfactory accuracy the most relevant environmental quantities for the application of interest, i.e., temperature, RH, pressure, CO_2_ and O_2_ concentrations, and can be expanded to monitor the soil moisture exploiting sensing systems as those presented in [36].

**Figure 3:**
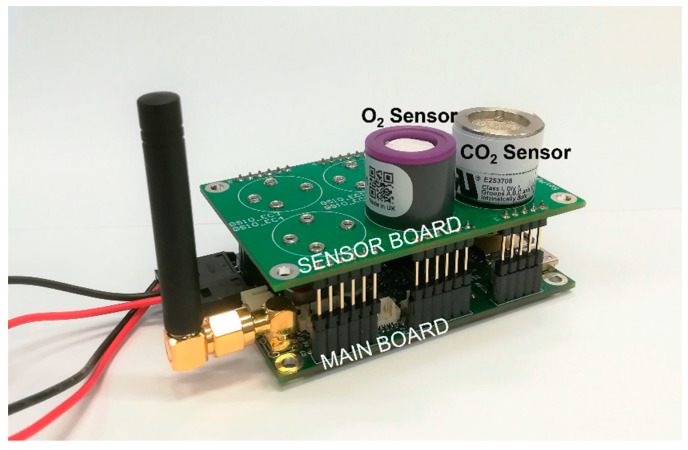
Designed node with the sensor board at the top and the main board at the bottom.

The gas sensors and their front-end electronics are hosted in a dedicated board called ‘sensor board’, which is connected to a second printed board, the ‘main board’, by means of some I/O pins of the MCU STM32L4Q5 by STMicroelectronics (Schiphol-Rijk, The Netherlands), as shown in Figure 3. This modular solution allows the adaptation of the node to different sensors, simply by re-designing the sensor board.

### 5.1. The PV Module

The polycrystalline silicon modules employed are BP Solar (Madrid, Spain) MSX-005F 0.5 W, composed of a series of 8 cells with active area A = 6.2 cm^2^. They feature nominal power P = 0.446 W, open circuit voltage V_OC_ = 4.6 V and short circuit current I_SC_ = 160 mA in standard test conditions (STC) 1.5 AMG.

### 5.2. The Sensor Board

**Figure 4:**
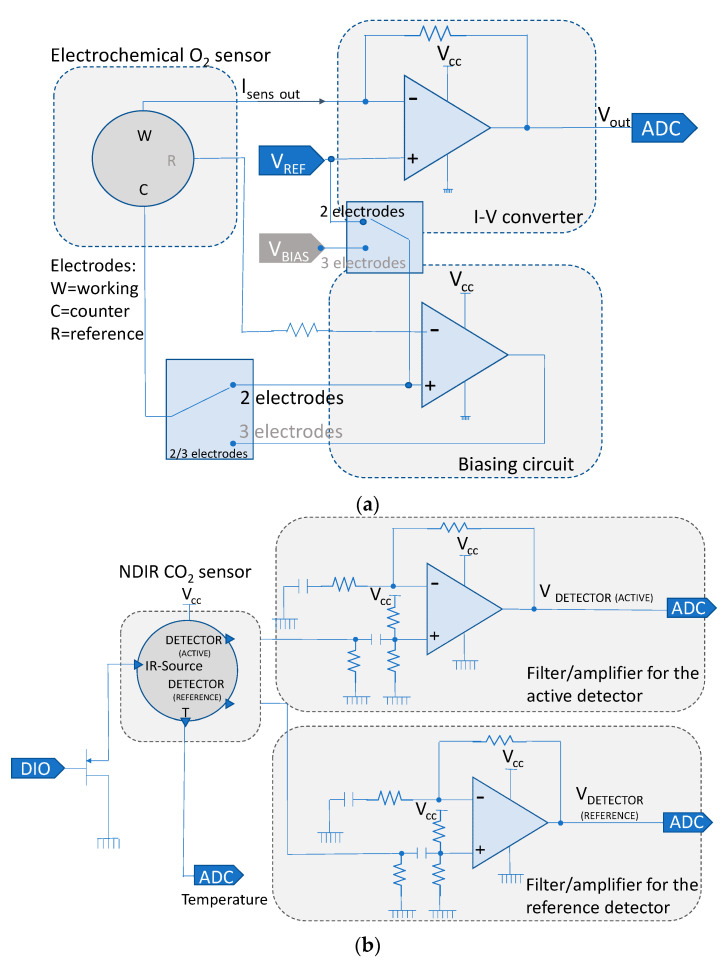
Front-end electronics of the (**a**) electrochemical O_2_ sensor and the (**b**) NDIR CO_2_ sensor.

To allow autonomous operations in the typical lightning treatments adopted in greenhouses, great care was devoted to the selection of low power sensing solutions, when possible. In detail, the autonomous sensing node embeds an amperometric electrochemical sensor O2-A3 (ALPHASENSE (Braintree, UK)) providing the measurement of the oxygen concentration with an accuracy lower than 1% and low drift for a period of 3 years. This is a two-electrode active sensor not requiring an external excitation and, as such, poses no problems from the power consumption point of view. Indeed, the power consumption related to the oxygen measurement is solely due to the front-end circuit, shown in Figure 4a, which is designed selecting ultra-low power analog components. It is composed of a current-to-voltage converter and a biasing circuit based on OP-AMPs. The critical point is the biasing circuit, designed to also allow the replacement of the selected sensor with a three-electrode electrochemical sensor, that must always be powered to avoid long transients related to the attainment of the chemical steady state. When using the two-electrode electrochemical sensor, the front-end exploits a regulated voltage V_REF_ = 1.25 V obtained from an ultra-low power reference circuit (quiescent current < 2 µA). In the three-electrode electrochemical sensor, the front-end not only exploits the regulated voltage reference V_REF_, but also another voltage reference derived from an external DAC at 12 bit. The overall current absorption of the front-end supplied with 3.3 V is <4 µA in the two-electrode case and <54 µA in the three-electrode configuration.

The main issue in the design of an autonomous node is the embedding of a CO_2_ sensor, in fact the most robust measurement solutions for carbon dioxide are based on optical techniques exploiting the CO_2_ absorption in the IR spectrum, which requires optical sources characterized by large power consumptions. In particular, the selected sensor is an IR21GM (SGX Sensortech (Neuchatel, Switzerland)), a non-dispersive-infrared sensor (NDIR) operating with a pulsed power source at 4 Hz, 50% duty cycle, 3.3 V with a mean current absorption of 26 mA. It embeds a LM60 internal temperature sensor (Texas Instruments (Dallas, TX, USA)) in order to perform temperature correction, absorbing about 100 µA. Moreover, this sensor requires a warm-up time of about 20 s and its response time is 20 s; therefore, to obtain a meaningful CO_2_ concentration reading, a power-on period longer than 40 s is needed. The output of the sensor is a voltage signal needing the A/D conversion of 1 s time window and digital processing to obtain the measurement result. The schematic of the front-end electronics for the CO_2_ sensor is shown in Figure 4b: the consumption of the conditioning circuit (amplifiers, filters and output voltage adjustment) is <200 µA.

### 5.3. The Main Board

The ‘main board’ hosts the MCU and the other needed components, i.e., the LoRa low power transceiver module RFM95x (Hoperf (Shenzhen, China)) equipped with a 2 dBi gain λ/8 antenna by means of a SMA connector soldered on the PCB, the nano-power DC-DC buck converter and boost charger module (CJMCU-2557) based on the BQ25570 integrated circuit (IC) (Texas Instruments (Dallas, TX, USA)), an environmental monitoring MEMS (Micro Electro-Mechanical Systems) sensor, the SWD (Serial Wire Debug) STMicroelectronics proprietary programming interface and the UART (Universal Asynchronous Receiver Transmitter) TX-RX interface.

For the measurement of temperature, pressure and RH, we used the BME280 (Bosch, Stuttgart, Germany), which is a low power MEMS with digital output (I^2^C (Inter-Integrated Circuit) or SPI (Serial Peripheral Interface)). The sensor can be powered with a voltage in the [1.71 V, 3.6 V] range and can provide 1 reading/s of the three quantities with a current absorption of 3.6 µA for the sensor reading (the average of 5 consecutive readings is taken, for a whole duration of 5 s), 0.1 µA in sleep mode and 100 µA for the I^2^C communication bus, ensuring 3% accuracy and 1 s response time for RH, 1.7 hPa accuracy for pressure and 1.2 °C accuracy for temperature.

The STM32L4Q5 with some active peripherals (i.e., I^2^C, SPI, ADC, internal voltage reference, the needed GPIOs, two timers) sinks in run mode approximately 500 µA, whilst in sleep mode its current absorption drops to 5 µA. It requires power supply in the [1.71 V, 3.6 V] range, which is compliant with the requirements.

The LoRa module is programmed according to the following radio settings: transmitting frequency 868 MHz, output power 14 dBm, coding rate (CR) 4/5, spreading factor (SF) 7 and bandwidth (BW) 125 kHz. The transceiver is powered at 3.3 V and in transmitting mode at SF 7 the current absorption reaches 80 mA, after the transmission, the device enters sleep mode with an absorption lower than <1 µA.

The nano-power commercial battery management system used in the node is the BQ25570, mounted in the CJMCU-2557 board. It integrates both the features of the boost charger and of the buck converter in such a way to extract power in the order of µW to mW and to provide a regulated voltage supply used to power up the sensor node.

It has quiescent current in the nA range and requires minimum DC input voltage of 100 mV, which makes this IC extremely suitable for those applications where energy provisioning is sporadic or discontinuous, as in in the case of harvesting from indoor artificial lights. Specifically, the boost charger manages the charging and the protection tasks of a rechargeable Li-Po battery, 3.7 V 720 mAh, employed as a power reserve for the designed system. The battery overcharging and undercharging protection is performed by the battery threshold control unit, adequately fixing two programmable voltage thresholds using external resistors. The buck converter provides a regulated voltage supply to the load, adjusted by a programmable voltage divider. Furthermore, the load is disconnected from the BMS when the voltage at the storage element falls below a pre-set value in order to avoid excessive drainage. In this work, the voltage directly delivered to the load is about 3.3 V whereas the Li-Po battery is used as a power reserve.

The CJMCU-2557 module exploits the maximum power point tracking (MPPT) capability of the BQ25570 IC and sets, in hardware, the working voltage of the energy source to 80% of its open circuit voltage (V_OC_), adapting this value with a period of 16 s. This feature aims to optimize energy harvesting from PV modules, since it theoretically corresponds to the maximum power transfer condition for these devices.

### 5.4. Node Operations

The MCU is programmed taking into account low power programming techniques, providing a sleep routine to periodically activate the MCU only for a limited time interval functional to the accomplishment of the firmware instructions. This timing can be varied according to different experimental settings: in the presented tests, we can consider a run mode of approximately 120 s every hour. In order to avoid extra power consumption during the MCU sleep, one digital I/O port of the MCU is used to power the CO_2_ sensor front-end, switching it on for the time needed to get a stable measurement (60 s). When the MCU is in run mode it samples the 3 sensors, processes the data and transmits via LoRaWAN the samples collected during every 8-h period, fulfilling the monitoring task of 24 readings per day.

Each packet has a payload of 80 bytes, organized as 10 bytes per hour with 2 bytes of information for each one of the 5 measured physical quantities (i.e., temperature, RH, pressure, CO_2_ and O_2_ concentrations). The transmitted packets are sent to three LoRaWAN gateways distributed in the building of the Department of Information Engineering and Mathematics, University of Siena, Siena, Italy. The gateways manage the redirection of the packets to a LoRaWAN server and then the received data are stored in a MySQL database making them available on the Internet. The decision to use the lowest CR and SF is dictated by the need to meet the low consumption requirement. Indeed, CR = 4/5 allows the shortest time on air (ToA), and consequently the lowest power consumption, at the expense of slightly worse error correction at the reception. Similarly, SF = 7 has the shortest ToA (~164 ms for 80 bytes payload size + 13 bytes overhead size) and the longest transmittable payload, with the drawback of the link margin reduction and the packet loss increase. However, these are relevant aspects especially in critical scenarios characterized by high noise and strong signal attenuation, which is not the case of a greenhouse deployment, where we are supposed to have enough radio coverage. For this reason, we decided to favor the optimization of the power management without employing more reliable but also more consuming solutions.

## 6. Experimental Results

In all the tests described hereafter, the PV module was placed horizontally in the central axis of the lamp at a vertical distance of 10 cm, where the LED lighting is nominally uniform.

### 6.1. Solar Cell Characterization

**Figure 5:**
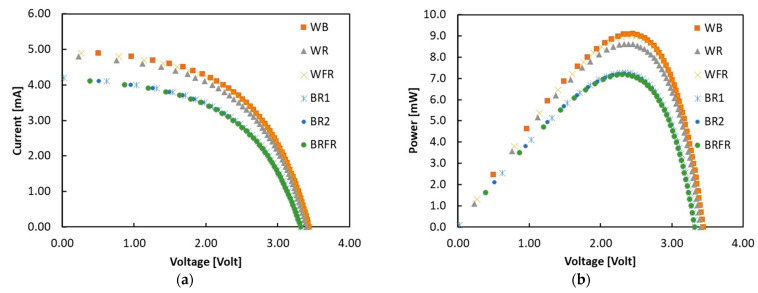
(**a**) I-V and (**b**) P-V characteristics measured in each lighting spectrum with the polycrystalline silicon photovoltaic module.

Figure 5 shows the I-V and P-V characteristics measured in each lighting condition given in Section 4 with the polycrystalline silicon photovoltaic module.

Table 1 summarizes the main photovoltaic parameters measured for each curve: short circuit current I_SC_, open circuit voltage V_OC_, fill factor (FF) and efficiency. The ratio between the peak power voltage, V_MAX_, and V_OC_, is also shown. Note that in all cases, this value is about 70%, lower than the 80% value used by the MPPT system. This effect is related to the low FF, in turn mainly due to the role of passive resistances smothering the I-V curves, a typical effect for silicon in low intensity conditions. The efficiency of the photovoltaic module is in the [5.4%, 6.4%] range, with the highest values in the case of BRFR. This is in line with the fact that red and far-red light components are corresponding to the range where the spectral response of the polycrystalline silicon, peaked at 940 nm, is higher, as discussed in [37].

**Figure 6:**
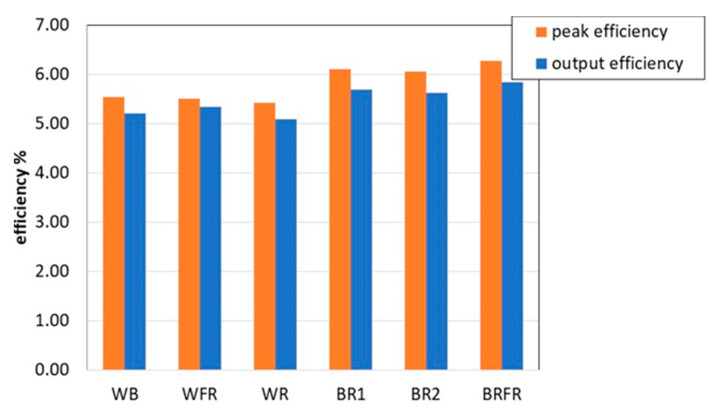
Comparison of the PV efficiency at the operating point (0.8 V_OC_) and at the peak point (0.7 V_OC_).

Since the BQ25570 sets the working voltage of the energy source to 0.8 V_OC_ whilst the maximum power point derived from the characterization corresponds to 0.7 V_OC_, the photovoltaic parameters of Table 1 are reformulated and presented in Table 2. In detail, V_MAX_ and P_MAX_ are the voltage and the extracted power at the peak point corresponding to 0.7 V_OC_, while V_OP_ and P_OP_ are the voltage and the extracted power at the operating point 0.8 V_OC_. The efficiencies in the two operating conditions are plotted in Figure 6.

### 6.2. Node Tests

Two test campaigns were performed using the sensor node described in Section 5. The node was tested under the six lighting protocols presented in Section 4 for 1 day with a 20 hd^−1^ photoperiod. The MCU was programmed to work in sleep mode, waking up every 1 h to sample the sensors and transmitting the collected data every 8 h (3 times per day). The goal of the tests was to prove the energy self-sufficiency of the proposed architecture in a realistic deployment, experimentally validating the characterization of the polycrystalline Si panel.

**Figure 7:**
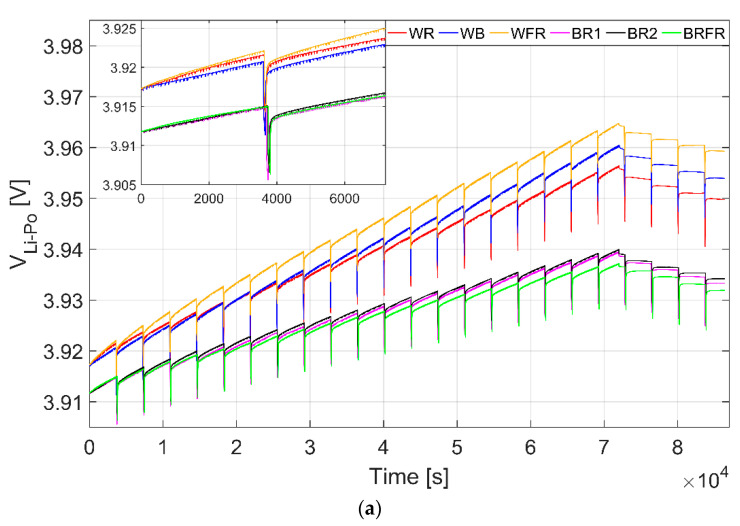

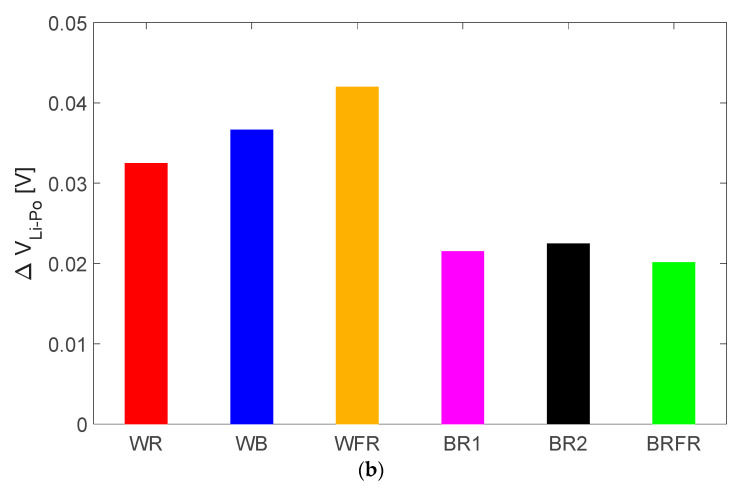
(**a**) Li-Po battery voltage, V_Li–Po_, trends during 24 h tests with a photoperiod of 20 hd^−1^ employing six light spectra: WR (in red), WB (in blue), WFR (in yellow), BR1 (in magenta), BR2 (in black) and BRFR (in green). In the inset, the V_Li–Po_ trends during the first 7200 s are reported. (**b**) Difference between the battery voltage level at the end and at the beginning of the test, (ΔV_Li–Po_), under the six light spectra.

The voltage trend of the rechargeable Li-Po battery (i.e., V_Li–Po_) was measured during the overall test, during which the node powered by the PV module acquired data from the sensors each hour and transmitted data packets 3 times per day. In particular, the most energy-consuming activities are on one hand the LoRa radio transmission phases, requiring working currents with peaks up to 80 mA but for a reduced time (~164 ms for each transmission, ~492 ms during the whole day). The corresponding absorption peaks cannot be seen in the data reported in Figure 7a due to their short duration and to the low pass filtering of the measurement system. On the other hand, the sampling of the CO_2_ sensor, which is the most onerous activity performed by the node since it requires working currents of ~26 mA for a total span of 1440 s (60 s for each sensor reading, repeated 24 times per day), causes the absorption peaks which can be noticed in Figure 7a.

A first set of tests was performed using the solar simulator programmed to emit the three light spectra containing the 5700 K white component (i.e., WB, WR, WFR) and always giving the same amount of total PPFD (i.e., 155 µmol/m^2^ s). The second set of tests was performed using the commercial horticulture lamp programmed in order to give BR1, BR2 and BRFR spectra with the same amount of total PPFD (i.e., 155 µmol/m^2^ s).

The Li-Po battery voltage trends are shown in Figure 7a, together with an inset reporting the same plots in the first 7200 s to highlight the charging slopes. The voltage behavior during the 24 h is composed of 20 increasing segments corresponding to the charging periods and 4 decreasing parts which are the hours of battery discharge (i.e., in the absence of light). As already stated, the equally spaced vertical lines are the spikes caused by the CO_2_ sensor readings which cause the lowering of the battery voltage for 60 s. In Figure 7b, the differences between the battery voltage level at the end and at the beginning of the six tests (ΔV_Li–Po_) are reported: they are equal to 36.65 mV, 32.47 mV, 41.99 mV, 20.14 mV, 21.55 mV and 22.46 mV, respectively, for WB, WR, WFR, BRFR, BR1 and BR2 spectra. They show that a positive balance between battery charging and discharging is reached and that the self-sufficiency of the system is achieved with all the tested spectra.

In the tests containing the white light, a higher ΔV_Li–Po_ is attained. This can be justified considering that the incident powers P_inc_ are higher, although in general the efficiencies are lower than the ones achieved with the three spectra not exploiting the W component. Among the WB, WR and WFR spectra, the higher ΔV_Li–Po_ is the one obtained with the addition of FR light, thus validating the outcome derived from the cell characterization. Indeed, the three spectra are obtained by adding the same amount of µmol/m^2^ s for B, R and FR components and, despite the fact that the blue light emissions carry more energy, the best result is obtained with the FR light, due to the maximum efficiency of the polycrystalline Si panel in this range. Concerning the other three spectra, it can be seen that the ΔV_Li–Po_ is quite similar, in accordance with the P_OP_ and the operative efficiencies derived from the characterization. Furthermore, the fact that the highest efficiency at the operating point is in the BRFR case is justified by the better response of polycrystalline silicon in the FR range.

**Figure 8:**
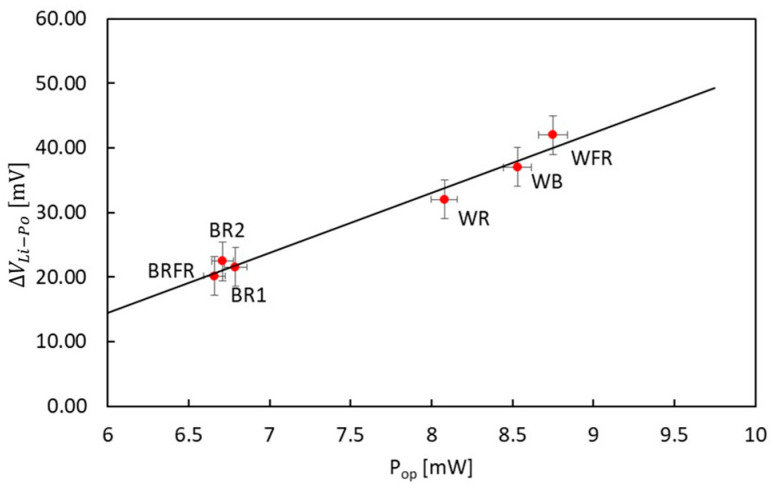
ΔV_Li–Po_ vs. power output delivered by the PV module, P_OP_, in different illumination conditions. Best fit shows a linear trend. Error on y-axis is fixed at 3 mV while error on x-axis is 1% of P_OP_.

The relation between P_OP_ and ΔV_Li–Po_ is shown in Figure 8 and the best fit shows a linear trend. The results of the six tests match the fitting well, especially the WB, WR and WFR spectra which have higher P_inc_ and consequently higher P_OP_. At lower P_OP_, the results are still consistent with the fitting and the slight variations from the expected results (modelled as a 3 mV error for the y-axis and 1% of P_OP_ error for the x-axis) can be linked to external factors affecting the battery charge, especially in the presence of low operating powers and ΔV_Li–Po_ as in the case study.

Moreover, the performance of the node can be extended beyond by resorting to higher PPFDs commonly employed in greenhouse deployments.

In Table 3, the daily energy balance of the system is reported, distinguishing the energy consumption contributions of the different parts composing the system. One reading of the sensors per hour and one data transmission every 8 h are assumed, while the current absorption of the BQ25570 is considered negligible with respect to the other contributions (in the order of hundreds of nA according to [38]). The maximum and minimum energies harvested by the PV panel during the 1-day tests are also reported, respectively, under WFR and BRFR lighting protocols. As expected, the most energy-consuming activity of the node is the CO_2_ sensor reading; therefore, the number of readings performed in a day is the critical factor affecting the energy self-sufficiency of the node.

According to the table, a surplus of energy is obtained (i.e., 346.8158 J in the minimum harvested energy case and 497.2958 J in the maximum harvested energy case), which determines a net charge of the Li-Po battery. This implies that more frequent data collection and packet transmissions would be possible with the actual harvested power with respect to those performed in the presented tests. Indeed, the maximum number of sensor readings and data transmissions per hour still guaranteeing the self-sufficiency of the node is three in the case of the minimum harvested energy and four in the case of the maximum harvested energy.

However, in practice this estimate may be affected by additional losses related to non-ideality of the system which reduce the actual energy surplus. Assuming with a conservative approach that the surplus is halved, it follows that the maximum realistic number of sensor readings and transmissions per hour is two in both cases, hence it is approximately double compared with the protocol adopted during the tests.

Given the fact that in this application timely data are not required, we assumed one measurement per hour to be an adequate sampling frequency for these preliminary tests.

## 7. Conclusions

The aim of this paper was to investigate the possibility of realizing an autonomous LoRaWAN-based wireless monitoring system for greenhouses. The designed sensor node exploits a polycrystalline silicon photovoltaic module illuminated by six lighting treatments commonly used in indoor horticulture. The designed sensor node exploits the PV module, a Li-Po battery and a harvesting system based on a nano-power boost charger buck converter in charge of extracting power from the indoor light, making it available for the node operations. A MCU deals with the sampling of the sensors and the data transmission via LoRa technology. Great attention was required in the design phase in order to create low power sensing circuits and strategies because of the low power extracted from the PV panel (in the mW range). In this sense, the most challenging task was the integration within the node of gas monitoring sensors such as the CO_2_ sensor, which is the most energy-consuming since it absorbs ~26 mA and requires reading times exceeding 40 s. The commercial solar cell was characterized under the six different spectral compositions obtained using two light sources, a custom designed solar simulator and a commercial lamp used for horticulture. The field tests in the six different conditions in which the node was monitored for one day each, proved the self-sufficiency of the node. Moreover, charging appears to be linearly correlated with the module power output. Best working conditions are achieved when white light is combined with a far-red component best matching the spectral response peak of the polycrystalline silicon. This makes the proposed sensor node a valid solution for energy-autonomous environmental monitoring inside greenhouses, performing energy harvesting from indoor light sources with narrow-band spectral components.

A future development can be the implementation of a customized Li-Po battery charging system tailored to the application.

## Figures and Tables

**Table 1:** PV parameters extracted from the I-V and the P-V characteristics in the different lighting conditions.

	WB	WR	WFR	BRFR	BR1	BR2
P_inc_ [W/m^2^]	33.00	32.00	33.00	23.00	24.00	24.00
I_SC_ [mA]	5.00	5.00	5.00	4.20	4.30	4.30
V_OC_ [V]	3.44	3.40	3.44	3.32	3.33	3.33
V_MAX_ [V]	2.46	2.33	2.44	2.31	2.35	2.33
V_MAX_/V_OC_ %	72.00	68.00	71.00	70.00	71.00	70.00
P_MAX_ [mW]	9.09	8.62	9.02	7.16	7.27	7.22
FF %	53.00	52.00	53.00	52.00	51.00	52.00
Efficiency_max_ %	5.55	5.43	5.51	6.28	6.11	6.07

**Table 2:** PV parameters at the operating point (0.8 V_OC_) and at the peak point (0.7 V_OC_).

	WB	WR	WFR	BRFR	BR1	BR2
P_inc_ [mW/m^2^]	3.30	3.20	3.30	2.30	2.40	2.40
V_OC_ [V]	3.44	3.40	3.44	3.32	3.33	3.33
V_MAX_ [V]	2.46	2.33	2.44	2.31	2.35	2.33
V_OP_ [V]	2.75	2.72	2.75	2.66	2.66	2.66
P_MAX_ [mW]	9.09	8.62	9.02	7.16	7.27	7.22
P_OP_ [mW]	8.53	8.08	8.75	6.66	6.79	6.71
Efficiency_max_ %	5.55	5.43	5.51	6.28	6.11	6.07
Efficiency*_OP_* %	5.21	5.09	5.35	5.84	5.70	5.64

**Table 3:** Daily energy balance of the system during the node operation, considering one reading of the sensors per hour and one data transmission every 8 h. The maximum and minimum harvested energies are computed, respectively, under WFR and BRFR lighting protocols.

	Component	Energy per Day [J]
Absorbed	O_2_ (front-end) CO_2_ (front-end) CO_2_ (sensor) BME280 (sensor) I^2^C bus STM32L4Q5 (run mode) STM32L4Q5 (stop mode) RFM95x (run mode) RFM95x (sleep mode) TOT	1.1405 0.9504 124.0272 0.0014 0.0396 4.7520 1.3781 0.1299 0.2851 132.7042
Harvested	PV module	max 630.0000
		min 479.5200

## Data Availability

Not applicable.
